# Bone Pain and Survival Among Patients With Metastatic, Hormone-Sensitive Prostate Cancer: A Secondary Analysis of the SWOG-1216 Trial

**DOI:** 10.1001/jamanetworkopen.2024.19966

**Published:** 2024-07-09

**Authors:** Georges Gebrael, Yeonjung Jo, Umang Swami, Melissa Plets, Chadi Hage Chehade, Arshit Narang, Shilpa Gupta, Zin W. Myint, Nicolas Sayegh, Catherine M. Tangen, Maha Hussain, Tanya Dorff, Primo N. Lara, Seth P. Lerner, Ian Thompson, Neeraj Agarwal

**Affiliations:** 1 Huntsman Cancer Institute at the University of Utah, Salt Lake City; 2 SWOG Statistics and Data Management Center, Seattle, Washington; 3 Cleveland Clinic Taussig Cancer Institute, Cleveland, Ohio; 4 Department of Internal Medicine–Division of Medical Oncology, University of Kentucky, Lexington; 5 Fred Hutchinson Cancer Research Center, Seattle, Washington; 6 Feinberg School of Medicine, Northwestern University, Chicago, Illinois; 7 City of Hope Comprehensive Cancer Center, Duarte, California; 8 University of California, Davis Comprehensive Cancer Center, Sacramento; 9 Baylor College of Medicine, Houston, Texas; 10 University of Texas Health Science Center at San Antonio, San Antonio

## Abstract

**Importance:**

The presence of bone pain is significantly associated with worse overall survival (OS) in patients with castration-resistant prostate cancer. However, there are few data regarding bone pain and survival outcomes in the context of metastatic, hormone-sensitive prostate cancer (MHSPC).

**Objective:**

To compare survival outcomes among patients with MHSPC by presence or absence of baseline bone pain at diagnosis.

**Design, Setting, and Participants:**

This post hoc secondary analysis, conducted from September 1 to December 31, 2023, used patient-level data from SWOG-1216, a phase 3, prospective randomized clinical trial that enrolled patients with newly diagnosed MHSPC from 248 academic and community centers across the US from March 1, 2013, to July 15, 2017. All patients in the intention-to-treat population who had available bone pain status were eligible and included in this secondary analysis.

**Interventions:**

In the SWOG-1216 trial, patients were randomized (1:1) to receive either androgen deprivation therapy (ADT) with orteronel, 300 mg orally twice daily (experimental group), or ADT with bicalutamide, 50 mg orally daily (control group), until disease progression, unacceptable toxic effects, or patient withdrawal.

**Main Outcomes and Measures:**

Overall survival was the primary end point; progression-free survival (PFS) and prostate-specific antigen (PSA) response were secondary end points. Cox proportional hazards regression models were used for both univariable and multivariable analyses adjusting for age, treatment type, Gleason score, disease volume, Zubrod performance status, and PSA level.

**Results:**

Of the 1279 male study participants, 301 (23.5%) had baseline bone pain at MHSPC diagnosis and 896 (70.1%) did not. Bone pain status was unavailable in 82 patients (6.4%). The median age of the 1197 patients eligible and included in this secondary analysis was 67.6 years (IQR, 61.8-73.6 years). Compared with patients who did not experience bone pain, those with baseline bone pain were younger (median age, 66.0 [IQR, 60.1-73.4] years vs 68.2 [IQR, 62.4-73.7] years; *P* = .02) and had a higher incidence of high-volume disease (212 [70.4%] vs 373 [41.6%]; *P* < .001). After adjustment, bone pain was associated with shorter PFS and OS. At a median follow-up of 4.0 years (IQR, 2.5-5.4 years), patients with bone pain had median PFS of 1.3 years (95% CI, 1.1-1.7 years) vs 3.7 years (95% CI, 3.3-4.2 years) in patients without initial bone pain (adjusted hazard ratio [AHR], 1.46; 95% CI, 1.22-1.74; *P* < .001) and OS of 3.9 years (95% CI, 3.3-4.8 years) vs not reached (NR) (95% CI, 6.6 years to NR) in patients without initial bone pain (AHR, 1.66; 95% CI, 1.34-2.05; *P* < .001).

**Conclusions and Relevance:**

In this post hoc secondary analysis of the SWOG-1216 randomized clinical trial, patients with baseline bone pain at MHSPC diagnosis had worse survival outcomes than those without bone pain. These data suggest prioritizing these patients for enrollment in clinical trials, may aid patient counseling, and indicate that the inclusion of bone pain in prognostic models of MHSPC may be warranted.

**Trial Registration:**

ClinicalTrials.gov Identifier: NCT01809691

## Key Points

**Question:**

Is baseline cancer-related bone pain associated with survival outcomes in patients with metastatic, hormone-sensitive prostate cancer (MHSPC) receiving systemic androgen deprivation therapy combined with a first- or second-generation androgen receptor pathway inhibitor?

**Findings:**

In this post hoc secondary analysis of the SWOG-1216 randomized clinical trial of 1279 patients with MHSPC, patients with bone pain at diagnosis had significantly shorter overall and progression-free survival compared with those who did not have bone pain.

**Meaning:**

The results may aid patient counseling and support the inclusion of bone pain in prognostic models of MHSPC and prioritization of patients with bone pain for enrollment in clinical trials.

## Teaser

This secondary analysis of a randomized clinical trial among patients with metastatic, hormone-sensitive prostate cancer examines the association of bone pain at diagnosis with overall and progression-free survival.

## Introduction

Overall survival (OS) among patients with metastatic, hormone-sensitive prostate cancer (MHSPC) receiving androgen deprivation therapy (ADT) intensification has significantly improved, reaching a median of 81.1 months.^1^ The currently recommended therapy for MHSPC is ADT with androgen receptor pathway inhibitors with or without docetaxel.^2^ Establishing baseline biomarkers that can reliably predict survival outcomes could facilitate more personalized treatment approaches. For instance, bone metastases contribute to a decline in quality of life through the induction of skeletal-related events and pain, impacting patients’ physical, emotional, and functional well-being.^3^ Multiple studies have consistently demonstrated that the presence of cancer-related bone pain in individuals with metastatic, castration-resistant prostate cancer (MCRPC) is associated with poorer prognosis.^4,5^ However, in the context of MHSPC, there are limited data on the effects of bone pain on survival outcomes. Hence, we sought to investigate the association between bone pain at MHSPC diagnosis and survival outcomes using patient-level data from the SWOG-1216 phase 3 randomized clinical trial.

## Methods

In the primary SWOG-1216 trial (NCT01809691), a multicenter trial funded by the National Cancer Institute (NCI) and conducted from March 1, 2013, to July 15, 2017, patients from 248 academic and community centers across the US with newly diagnosed MHSPC were randomized in a 1:1 ratio to receive either ADT with orteronel, 300 mg orally twice daily, in the experimental arm or ADT with bicalutamide, 50 mg orally daily, in the control arm until disease progression, unacceptable toxic effects, or patient withdrawal (the trial protocol is in Supplement 1). The primary outcome was OS. The methods and primary findings of the trial were detailed in a prior publication.^1^ The trial was approved by the NCI’s central institutional review board and complied with international Good Clinical Practice and the Declaration of Helsinki,^6^ with all participants giving written consent. This post hoc secondary analysis of the SWOG-1216 trial was conducted from September 1 to December 31, 2023. The current study adhered to the reporting guidelines outlined in the Consolidated Standards of Reporting Trials (CONSORT).

All patients in the intention-to-treat population of the original trial who had available bone pain status were eligible for this secondary analysis (eFigure in Supplement 2). In this secondary analysis, baseline bone pain was categorized based on the presence or absence of bone pain. The presence of bone pain (yes or no) was collected before registration (ie, at baseline and per the treating physician’s clinical assessment). Overall survival, defined as the time from randomization to death from any cause, was the primary end point. Key secondary end points were progression-free survival (PFS), defined as the time from randomization to biochemical, radiographic, or clinical progression (per Prostate Cancer Working Group 2 criteria) or death from any cause, and prostate-specific antigen (PSA) response. The PSA response rates were categorized at a 7-month landmark after random assignment into complete response (<0.2 ng/mL), partial response (0.2-4.0 ng/mL), and no response (>4.0 ng/mL) (to convert to μg/L, multiply by 1.0).

### Statistical Analysis

Baseline characteristics were summarized using medians and interquartile ranges (IQRs) for nonnormally distributed continuous variables and counts and percentages for categorical variables. Differences in baseline characteristics by bone pain status at MHSPC diagnosis were assessed with χ^2^ tests for categorical variables and Wilcoxon tests for continuous variables. Survival outcomes, including median PFS and OS, were calculated using Kaplan-Meier curves. Cox proportional hazards regression models were used for both univariable and multivariable analyses adjusting for variables including age, treatment type, Gleason score, disease volume, Zubrod performance status (score range, 0-4, with higher scores indicating poorer performance status), and PSA level, which was log_2_ transformed for normalization. Complete cases without any missing information were included in our multivariable model. We used a significance level of *P* <.05, and all hypotheses tested were 2-sided. R software, version 4.2.3 (R Project for Statistical Computing) was used for statistical analysis.

## Results

Of the 1279 male patients in the intention-to-treat population, 301 (23.5%) experienced baseline bone pain at MHSPC diagnosis while 896 (70.1%) did not. Bone pain status was unavailable for 82 patients (6.4%). For the 1197 included patients with available bone pain status, the median age was 67.6 years (IQR, 61.8-73.6 years); 585 patients (48.9%) had a high disease burden, and 1151 (96.2%) had a baseline Zubrod performance status of 0 or 1. Patients with baseline bone pain, compared with those without, were significantly younger at MHSPC diagnosis (median age, 66.0 years [IQR, 60.1-73.4 years] vs 68.2 years [IQR, 62.4-73.7 years]; *P* = .02); had significantly higher median PSA values at baseline (61.5 ng/mL [IQR, 16.4-272.3 ng/mL] vs 22.9 ng/mL [IQR, 9.1-81.5 ng/mL]; *P* < .001); had higher incidence of high-volume disease, defined as greater than minimal involvement of vertebrae, pelvic bones, and/or lymph nodes (212 [70.4%] vs 373 [41.6%]; *P* < .001); had higher incidence of liver metastasis (13 [4.3%] vs 14 [1.6%]; *P* = .01); and had a poorer Zubrod performance status (127 [42.2%] vs 684 [76.3%] were fully active; *P* < .001) (Table 1). However, patients with bone pain, compared with those without, had similar frequency of visceral metastasis (32 [10.6%] vs 112 [12.5%]; *P* = .45), frequency of Gleason score of 8 or higher (174 of 264 [65.9%] vs 534 of 865 [61.7%]; *P* = .25), and treatment assignment (158 [52.5%] vs 443 [49.4%] received orteronel; *P* = .40). At a median follow-up of 4.0 years (IQR, 2.5-5.4 years), the median OS for patients with baseline bone pain was 3.9 years (95% CI, 3.3-4.8 years) compared with not reached (NR) (95% CI, 6.6 years to NR) for patients without baseline bone pain (adjusted hazard ratio [AHR], 1.66; 95% CI, 1.34-2.05; *P* < .001). Similarly, the median PFS was 1.3 years (95% CI, 1.1-1.7 years) for patients with baseline bone pain and 3.7 years (95% CI, 3.3-4.2 years) for patients without baseline bone pain (AHR, 1.46; 95% CI, 1.22-1.74; *P* < .001) (Table 2 and the Figure). Among the 227 patients with an available PSA level at 7 months and with baseline bone pain, 105 (46.3%) had a complete PSA response at 7 months and 45 (19.8%) had no PSA response at 7 months compared with 504 (66.3%) and 64 (8.4%) of the 760 patients with available PSA levels and without baseline bone pain, respectively (*P* < .001).

**Table 1.** Baseline Characteristics and PSA Responses

Variable	Patients^a^		*P* value
	Presence of bone pain (n = 301)	Absence of bone pain (n = 896)	
Age, median (IQR), y	66.0 (60.1-73.4)	68.2 (62.4-73.7)	.02
Zubrod performance status			
Fully active	127 (42.2)	684 (76.3)	<.001
Restricted activity	141 (46.8)	199 (22.2)	
No work, ambulatory	27 (9.0)	12 (1.3)	
Limited self-care	5 (1.7)	0	
Unknown	1 (0.3)	1 (0.1)	
Gleason score ≥8, No./total No. (%)	174/264 (65.9)	534/865 (61.7)	.25
High-volume disease^b^	212 (70.4)	373 (41.6)	<.001
Visceral metastasis	32 (10.6)	112 (12.5)	.45
Liver metastasis	13 (4.3)	14 (1.6)	.01
PSA level at baseline, median (IQR), ng/mL	61.5 (16.4-272.3)	22.9 (9.1-81.5)	<.001
Treatment group			
Orteronel	158 (52.5)	443 (49.4)	.40
Bicalutamide	143 (47.5)	453 (50.6)	
PSA response at 7 mo, No./total No.			
Complete response	105/227 (46.3)	504/760 (66.3)	<.001
Confirmed partial response	77/227 (33.9)	192/760 (25.3)	
No response	45/227 (19.8)	64/760 (8.4)	

Abbreviations: IQR, interquartile range; PSA, prostate-specific antigen.

SI conversion factor: To convert PSA to μg/L, multiply by 1.0.

^a^ Data are presented as number (percentage) of patients unless otherwise indicated.

^b^ Defined as greater than minimal involvement of vertebrae, pelvic bones, and/or lymph nodes.

**Table 2.** Multivariable Analysis of PFS and OS

Characteristic	AHR (95% CI)^a^			
	PFS	*P* value	OS	*P* value
Bone pain				
No	1 [Reference]	NA	1 [Reference]	NA
Yes	1.46 (1.22-1.74)	<.001	1.66 (1.34-2.05)	<.001
Treatment				
Bicalutamide	1 [Reference]	NA	1 [Reference]	NA
Orteronel	0.56 (0.48-0.66)	<.001	0.87 (0.72-1.04)	.13
High-volume disease^b^	1.73 (1.48-2.04)	<.001	1.75 (1.42-2.14)	<.001
Gleason score ≥8	1.26 (1.07-1.48)	.005	1.21 (0.99-1.47)	.07
Log_2_-transformed PSA level	1.11 (1.08-1.15)	<.001	1.07 (1.03-1.11)	<.001
Zubrod performance status^c^				
0-1	1 [Reference]	NA	1 [Reference]	NA
≥2	1.67 (1.13-2.45)	.009	2.07 (1.37-3.13)	<.001
Age, per 1-y increase	1.00 (0.99-1.01)	.80	1.01 (1.00-1.02)	.08

Abbreviations: AHR, adjusted hazard ratio; NA, not applicable; OS, overall survival; PFS, progression-free survival; PSA, prostate-specific antigen.

^a^ Adjusted for age, treatment type, Gleason score, disease volume, Zubrod performance status, and log_2_-transformed PSA level.

^b^ Defined as greater than minimal involvement of vertebrae, pelvic bones, and/or lymph nodes. Low-volume disease was the reference category.

^c^ Possible score range, 0-4, with higher scores indicating poorer performance status.

**Figure.**
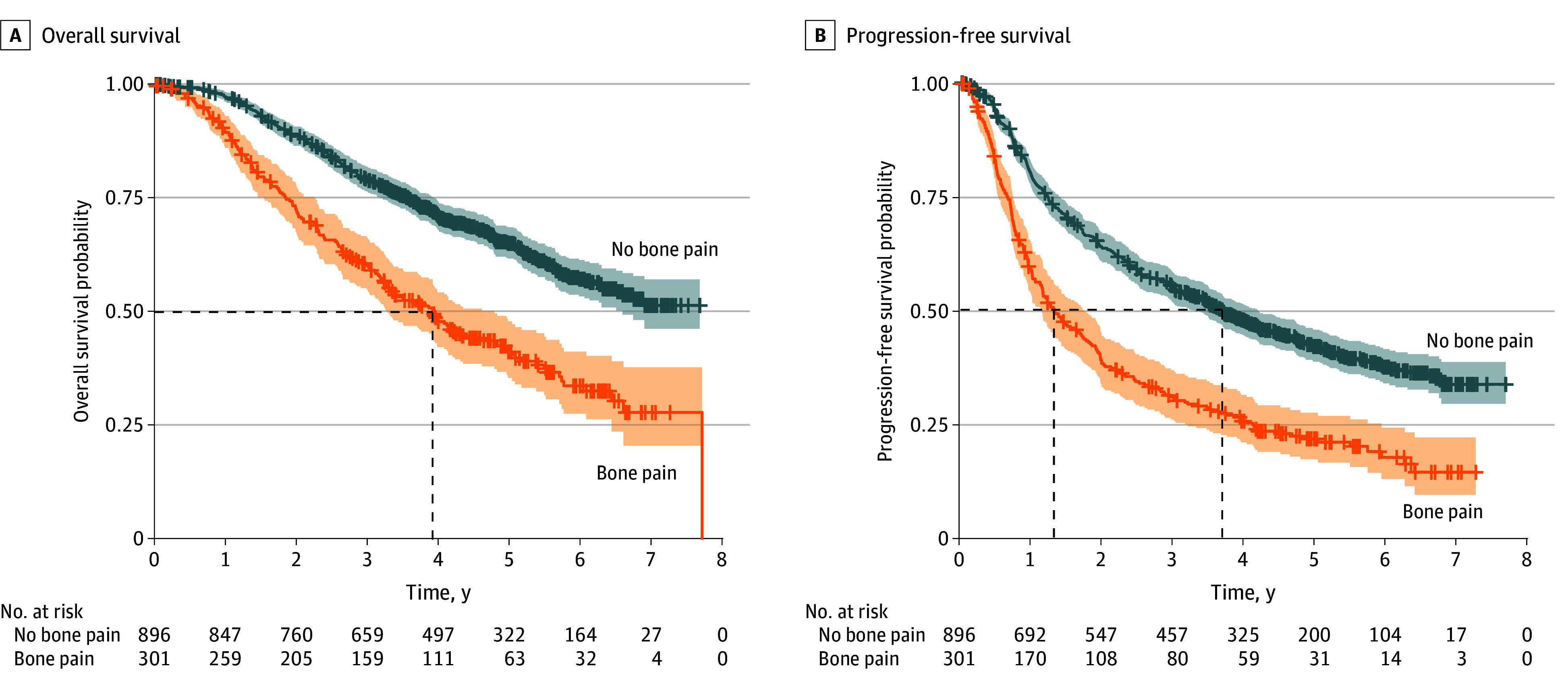
Kaplan-Meier Estimates of Overall Survival (OS) and Progression-Free Survival (PFS) by Bone Pain in the Overall Population Dashed lines indicate the median time participants experienced OS or PFS; shading represents 95% CIs.

## Discussion

In this post hoc secondary analysis of the SWOG-1216 randomized clinical trial, we found that patients presenting with bone pain at the time of MHSPC diagnosis and treated with ADT in combination with either bicalutamide or a novel androgen receptor pathway inhibitor (orteronel) had poorer OS and PFS than those without bone pain at diagnosis. Additionally, those with bone pain had more aggressive disease presentation and lower complete PSA response rates. In general, patient-reported outcomes, including pain, significantly impact clinical outcomes in patients with cancer.^7^ In patients with MCRPC, cancer-related pain and the use of opioid analgesics have served as prognostic determinants for unfavorable clinical progression independently of the treatment received.^4,8^ Similarly, pain has consistently been shown to be a factor associated with lower OS, notably in patients with MCRPC treated with abiraterone or enzalutamide.^9,10^ In the context of MHSPC, there are limited data from prospective trials regarding bone pain and survival outcomes. In the GETUG-15 trial investigating docetaxel with ADT, pain intensity was shown as one of the factors associated with reduced OS.^11^

A possible biological rationale underlying the association between bone pain and prostate cancer survival outcomes may stem from the presence of growth factors within the prostate tumor, notably nerve growth factor (NGF). Nerve growth factor has the capacity to modulate inflammatory and neuropathic pain conditions.^12^ Furthermore, NGF can trigger the activation of tropomyosin receptor kinase A (TrkA), which could initiate the proliferation and progression of prostate tumor cells through the interaction between TrKa and the androgen receptor, a pivotal component in prostate cancer proliferation.^13^ Therefore, NGF could mediate both pain and tumor growth.

### Limitations

The limitations of this secondary analysis of a prospective phase 3 trial include its post hoc nature. In addition, although orteronel significantly improved PFS, it did not improve OS and hence did not garner regulatory approval.^1^ Furthermore, the multivariable analysis in the current study did not account for synchronous vs metachronous disease status since this was not an established prognostic factor at the time when the original trial was conceptualized in 2011. However, bone pain was associated with poor outcomes regardless of the treatment arm in our current analysis and therefore may have implications in the management of MHSPC after external validation.

## Conclusions

This post hoc analysis of the SWOG-1216 phase 3 randomized clinical trial found that patients with MHSPC with baseline bone pain had worse survival outcomes than those without baseline bone pain. These results highlight the need to consider bone pain in prognostic modeling, treatment selection, patient monitoring, and follow-up and suggest prioritizing these patients for clinical trials and immediate systemic treatment initiation.

## Notes

Supplement 1.
Trial Protocol

Supplement 2. **eFigure.** CONSORT Diagram for the SWOG-1216 Trial

Supplement 3.
Data Sharing Statement
