# Supplementary material for: Bone Pain and Survival Among Patients With Metastatic, Hormone-Sensitive Prostate Cancer: A Secondary Analysis of the SWOG-1216 Trial
Source: JAMA Netw Open. 2024 Jul 9;7(7):e2419966. doi: 10.1001/jamanetworkopen.2024.19966 (PMC11234233; doi:10.1001/jamanetworkopen.2024.19966)
Supplement: Supplement 2. — eFigure. CONSORT Diagram for the SWOG-1216 Trial [file jamanetwopen-e2419966-s002.pdf]

Supplementary Online Content

Gebrael G, Jo Y, Swami U, et al. Bone pain and survival among patients with metastatic, hormone-sensitive prostate cancer: a secondary analysis of the SWOG-1216 trial. *JAMA Netw Open*. 2024;7(7):e2419966. doi:10.1001/jamanetworkopen.2024.19966

eFigure. CONSORT Diagram for the SWOG-1216 Trial

This supplementary material has been provided by the authors to give readers additional information about their work.

© 2024 Gebrael G et al. *JAMA Network Open*.

eFigure. CONSORT diagram for the SWOG-1216 trial.

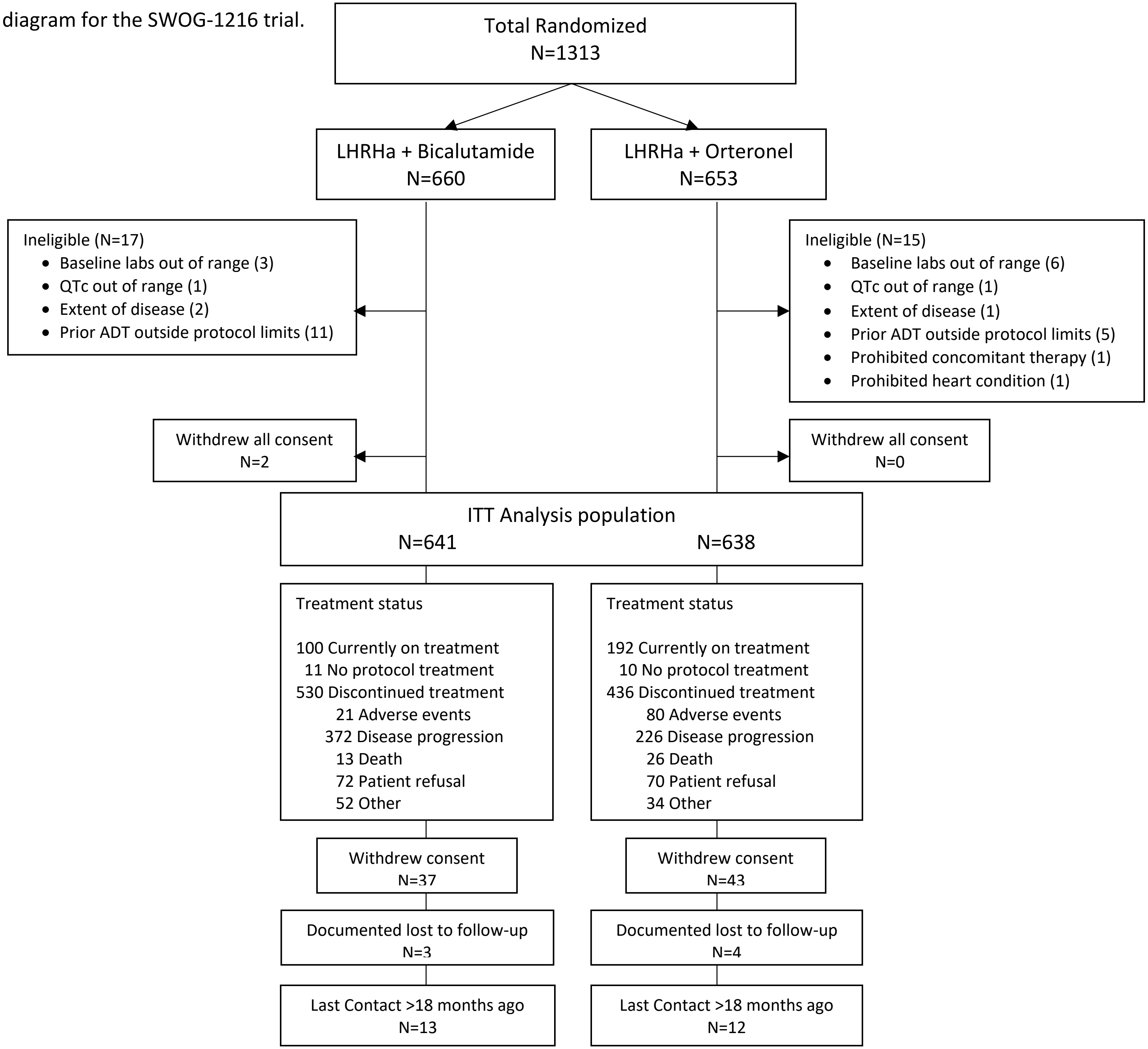

© 2024 Gebrael G et al. *JAMA Network Open*.
