# Supplementary material for: Bone Pain and Survival Among Patients With Metastatic, Hormone-Sensitive Prostate Cancer: A Secondary Analysis of the SWOG-1216 Trial
Source: JAMA Netw Open. 2024 Jul 9;7(7):e2419966. doi: 10.1001/jamanetworkopen.2024.19966 (PMC11234233; doi:10.1001/jamanetworkopen.2024.19966)
Supplement: Supplement 3. — Data Sharing Statement [file jamanetwopen-e2419966-s003.pdf]

Data Sharing Statement

Gebrael. Bone Pain and Survival Among Patients With Metastatic, Hormone-Sensitive Prostate Cancer. *JAMA Netw Open*. Published July 09, 2024.
doi:10.1001/jamanetworkopen.2024.19966

Data
Data available: No

Additional Information
Explanation for why data not available: This is a NCI funded SWOG trial. The data will appear in the public domain in the due course of time after the analysis of all pre-specified endpoints have been reported. However, we do not know exactly when this will happen.
